# Obstructive Sleep Apnea and Sleep Structure Assessed in Polysomnography and Right Ventricular Strain Parameters

**DOI:** 10.3390/brainsci12030331

**Published:** 2022-02-28

**Authors:** Piotr Macek, Małgorzata Poręba, Aneta Stachurska, Helena Martynowicz, Grzegorz Mazur, Paweł Gać, Rafał Poręba

**Affiliations:** 1 Department of Internal Medicine, Occupational Diseases, Hypertension and Clinical Oncology, Wroclaw Medical University, Borowska 213, 50-556 Wroclaw, Poland; piotr.macek@umw.edu.pl (P.M.); poreba1@wp.pl (M.P.); aneta.stachurska@umw.edu.pl (A.S.); helena.martynowicz@umw.edu.pl (H.M.); grzegorz.mazur@umw.edu.pl (G.M.); rafal.poreba@umw.edu.pl (R.P.); 2 Department of Paralympic Sports, Wroclaw University of Health and Sport Sciences, Witelona 25a, 51-617 Wroclaw, Poland; 3 Department of Population Health, Division of Environmental Health and Occupational Medicine, Wroclaw Medical University, Mikulicza-Radeckiego 7, 50-368 Wroclaw, Poland

**Keywords:** obstructive sleep apnea, right ventricle, sleep structure, strain

## Abstract

Our study aimed to assess functional, structural changes of the right ventricular using strain parameters and sleep structure using polysomnography in patients with obstructive sleep apnea (OSA). Our study group consisted of 43 patients, 29 men, 14 women. The mean age was 56.36 ± 14.77. All patients underwent full night polysomnography and transthoracic echocardiography. The right ventricular global longitudinal strain was measured by 2D speckle-tracking echocardiography. The prevalence of OSA (AHI ≥ 5) was 76.7% in the studied group. We observed a significant positive correlation between OAH and average free wall strain (r = 0.37), snore and mid-free wall strain (r = 0.34), average HR, and basal free wall strain (r = 0.34). Moreover, CSB was positively correlated with basal septal strain and mid septal strain (r = 0.36 and 0.42). In summary, among patients with sleep disorders, functional disorders of the right ventricle, assessed using the strain method, are partly observed.

## 1. Introduction

Obstructive sleep apnea (OSA) is a chronic sleep breathing disorder characterized by frequent episodes of collapsing of the upper airway during sleep [1]. It leads to an acute desaturation of arterial blood and arousal due to reopening of the airway and continued breathing. Clinically, the patients who suffer from OSA can complain of daytime sleeping, snoring, impaired concentration, morning headaches, and insomnia [2]. Moreover, OSA in some cases can cause arrythmias. There are few mechanisms including nervous system fluctuations, intermittent hypoxia [3]. Pollicina et al. indicated in their study the relationship between obstructive sleep apnea and neurodegenerative diseases with low cognitive performance and low memory. It has been indicated that CPAP treatment seems to improve cognitive defects [4].

The recurrent hypoxia and hypercapnia increase the risk of cardiovascular and cerebrovascular disorders caused by oxidative stress due to inadequate arterial blood oxygenation and metabolic demand [5].

OSA frequency in the general population affects at least 9–15% of middle-aged adults, and significantly, it is more common among men. Many factors increase OSA’s risk and severity, such as age, overweight, alcohol use and self-reported previous cardiovascular disorders [6]. One study indicated a higher risk of OSA in patients with rhinitis as Pace et al. showed that patients with non-allergic rhinitis with eosinophilia syndrome were at increased risk of OSA with respect to allergic rhinitis and healthy individuals [7].

Based on the American Academy of Sleep Medicine the sleep-related breathing disorders may be divided into obstructive sleep apnoea, central sleep apnoea with or without Cheyne-Strokes breathing pattern and sleep-related hypoventilation. These states can lead to increase pulmonary arterial pressure. The most common association of chronic obstructive pulmonary disease and OSA can cause pulmonary hypertension. The patients with OSA and pulmonary hypertension should be treated using CPAP [8].

Pulmonary hypertension is a hemodynamic and pathophysiological state that can be found among patients with OSA [9]. This disorder occurs among 20–40% of patients [10]. Moreover, 27–30% of individuals without left ventricular dysfunction or hypoxemic lung disease have PH [11]. The people with OSA and PH have a lower quality of life and higher mortality than those without PH [12]. There are two main reasons for PH. Pulmonary hypertension can be caused by dysfunction of the left ventricular. In this case, increasing pressure in the left atrium causes a destructive effect on the pulmonary vessels. Its consequences are remodelling of vessels and the presence of PH [13]. The other reason is the decreasing level of oxygen in the airway, which results in the contraction of the pulmonary vessels. For patients with OSA, episodes of oxygen desaturation during the night are characterized. The cumulative effect of hypoxia can lead to PH [14].

Furthermore, pulmonary hypertension in patients with OSA can lead to the development of right ventricular hypertrophy and dysfunction in more severe cases. Junfang et al. indicated in their studies that right ventricular diastolic dysfunction begins before developing heart failure and pulmonary hypertension in patients with OSA [15].

Echocardiography is a suitable, non-invasive method for structural and functional assessment of the heart. A few studies concerning echocardiographic changes in the right ventricular of patients with OSA were published in recent years [16,17,18].

Imaging techniques based on strain have been shown increasing clinical utility in assessing the heart chambers, especially for visualization of the right ventricular [19].

Our study aimed to assess functional, structural changes of the right ventricular using strain parameters and sleep structure using polysomnography in patients with obstructive sleep apnea. 

## 2. Materials and Methods

The study was performed in the Sleep Laboratory in the Department of Internal Medicine, Occupational Diseases, Hypertension, and Clinical Oncology at Wroclaw Medical University, Poland.

The patients were admitted to our Clinic due to arterial hypertension or obstructive sleep apnea diagnostics. Our study group consisted of 43 patients, 29 men, 14 women. The mean age was 56.36 ± 14.77.

Our study included individuals who declared their willingness to participate and fulfilled the following criteria: age above 18 years and clinical suspicion of obstructive sleep apnea or resistant arterial hypertension. Exclusion criteria were as follows: immature patients, inability to undergo polysomnography or echocardiography, intake of medicines affecting neuromuscular functioning or breath function, the presence of severe mental disorders, active malignancy, neurological disorders, and/or neuropathic pain, the co-existence of respiratory insufficiency or active inflammation.

The study was approved by the Ethics Committee of the Wroclaw Medical University in principles of the Declaration of Helsinki. The study was designed and described in accordance with the STROBE guidelines. The participants received all information about the study.

Full night polysomnography using Nox-A1 (Nox Medical, Reykjavik, Iceland) was performed among all patients in the Sleep Laboratory of the Department of Internal Medicine, Occupational Diseases, Hypertension and Clinical Oncology at the Wroclaw Medical University, Poland. Polysomnograms (PSGs) were scored in 30-s epochs and were classified based on standard criteria for sleep by the American Academy of Sleep Medicine 2013 Task Force. PSG outcomes included as follows: sleep latency, REM (rapid eye movement) latency, total sleep time, sleep efficiency, the ratio of N1 (sleep stage 1), N2 (sleep stage 2), N3 (sleep stage 3), and the stage of REM (rapid eye movement sleep stage) [20].

The day after polysomnography, echocardiography was performed. All patients underwent transthoracic echocardiography conducted by a single cardiologist. The echocardiogram was obtained in all precordial positions using 2-dimensional, M-mode, conventional, and tissue Doppler examination according to the American Echocardiography Association guidelines. Apical 4-chamber left parasternal long-axis (PLAX) and parasternal short-axis (PSAX), left parasternal RV inflow can provide images for the assessment of RV systolic and diastolic function and RV systolic pressure (RVSP). The right ventricular (RV) dimension is measured at end-diastole from a right ventricle–focused apical 4-chamber view. The right atrium dimension was also estimated in apical view. The left PSAX (left parasternal short axis) view demonstrates RVOT (RV outflow tract) at the pulmonic valve level. In contrast, the left PLAX (left parasternal long axis) view allows for the measurement of the proximal portion of the RVOT.

The right ventricular systolic function was calculated using several parameters, such as RIMP (right ventricular index of myocardial performance), TAPSE (tricuspid annular plane systolic excursion), and tissue Doppler-derived tricuspid lateral annular systolic velocity.

Assessment of RV diastolic function was performed using the pulsed Doppler of the tricuspid inflow, tissue Doppler of the lateral tricuspid annulus, and measurements of IVC size and collapsibility.

A strain is defined as a percentage change in myocardial deformation. The right ventricular global longitudinal strain (RVGLS) was measured by 2D speckle-tracking echocardiography, in which deformation of the right ventricular is determined by tracking speckles from frame to frame. It was calculated in the four-chamber view by speckle-tracking analysis. The RVGLS was obtained as a mean value of three segments of the RV free wall (basal, mid, and apical) and the intraventricular septum (basal, mid, and apical).

Statistical analyses were performed using the statistical package “Dell Statistica 13.1” (Dell Inc., Tulsa, OK, USA). The distribution of variables was checked by Lilliefors and W-Shapiro–Wilk tests. The *t*-test was used for the independent quantitative variables with the normal distribution. For variables with a distribution other than normal, the Mann–Whitney U-test was used for quantitative independent variables. For independent qualitative variables, the quadrate-square test of the highest reliability was used. Correlation and regression analysis was performed to determine the relationships between the variables studied. Parameters of the model obtained in the regression analysis were estimated using the least-squares method. The results on the level of *p* < 0.05 were assumed to be statistically significant.

## 3. Results

The mean age of all participants was 56.36 ± 14.77 years. Women constituted 32.7% (*n* = 14) of all the participants. The mean BMI was 26.71 ± 25.75 kg/m^2^. Diabetes and coronary artery diseases were diagnosed in 11.6% (*n* = 5) and 9.3% (*n* = 4) of the study patients, respectively. Hypertension was diagnosed in 60.5% (*n* = 26) patients. All clinical characteristics in the study group are included in Table 1.

The mean AHI was 27.09 ± 19.76. The prevalence of OSA (AHI ≥ 5) was 76.7% (*n* = 33) in the studied group. All polysomnography parameters are presented in Table 2.

Right ventricular echocardiography and right ventricular strain parameters are included in Table 3 and Table 4.

We observed statistically significant differences between patients with OBS (AHI ≥ 5) and without OBS considering average free wall strain (−32.64 vs. −27.17 *p* = 0.023), mild free wall strain (−33.71 vs. −28.30 *p* = 0.020), average septal strain (−20.24 vs. −15.93 *p* = 0.015), basal septal strain (−20.14 vs. −16.06 *p* = 0.030), mid septal strain (−20.57 vs. −16.48 *p* = 0.030), and apex septal strain (−20 vs. −15.24 *p* = 0.037), as shown in Table 5.

No statistically significant correlation was found between AHI and all right ventricular strain parameters. We observed a significant positive correlation between OAH and average free wall strain (r = 0.37), snore and mid-free wall strain (r = 0.34), average HR, and basal free wall strain (r = 0.34). Moreover, CSB was positively correlated with basal septal strain and mid septal strain, r = 0.36 and 0.42, respectively. The correlation between polysomnography and right ventricular parameters are included in Table 6.

**Figure 1:**
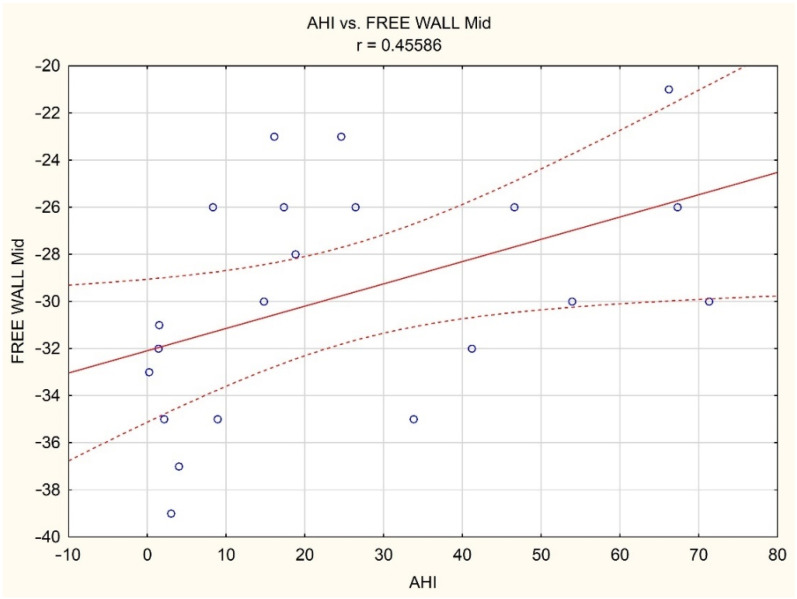
Correlation between AHI and RV mid free wall strain in the study subgroup with age below 60 years (red solid line—estimation curve, dashed lines—95% confidence interval of the estimation curve, small blue circles—individual cases).

In the study subgroup with age below 60 years, a positive correlation was observed between AHI and mid free wall strain (Figure 1). 

**Figure 2:**
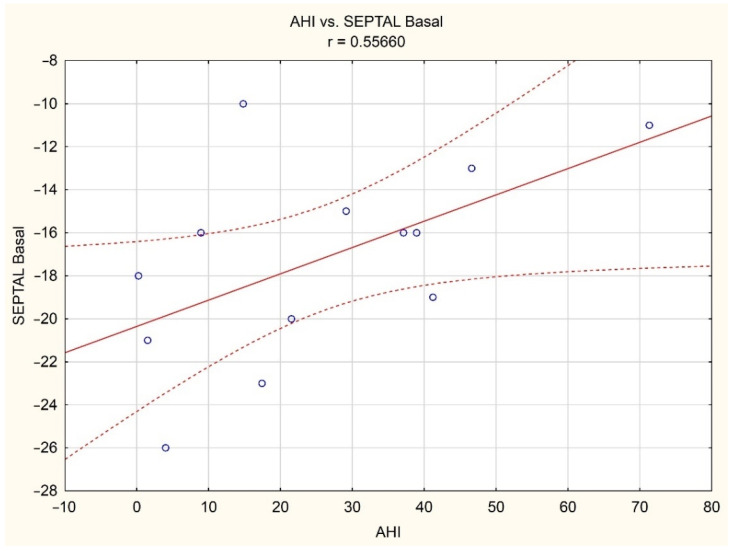
Correlation between AHI and RV basal septal strain in women subgroup (red solid line—estimation curve, dashed lines—95% confidence interval of the estimation curve, small blue circles—individual cases).

Moreover, we found a statistically significant correlation between AHI and basal septal strain (r = 0.56) only in the women subgroup (Figure 2).

## 4. Discussion

In our study, we showed statistically significant differences considering the severity of obstructive sleep apnea and average free wall strain, mid free wall strain, average, basal, mid, and apex septal strain. Based on correlation analysis, we have indicated that snore and mild free wall strain, OAH and average free wall strain, average hr and basal free wall strain, CSB and basal septal, and mid septal strain are correlated respectively.

Obstructive sleep apnea harms the cardiovascular system by mechanical, neurohumoral, and inflammatory mechanisms. Obturation of the respiratory tract cause during inspiration: decreased intrathoracic pressure, hypoxia, and arousal. The drop in intrathoracic increases left ventricular transmural pressure, which caused increased afterload and consequently venous return. The other effect contains right ventricular distention, a leftward shift of the interventricular septum, and decreased LV filling. The reduction in minute capacity results from the mechanism described [2]. Obstructive sleep apnea also causes repeated elevations in systemic blood pressure due to sleep arousal and the sympathetic nervous system’s hyperactivity [21]. Combining these two mechanisms may lead to insufficient oxygenation of the heart and predisposing the patient to coronary artery disease, arrhythmias, left ventricular hypertrophy, and sometimes heart failure [22,23,24]. The prevalence of PH in OSA depending on the methodology of the studies. The most recent research agrees on a prevalence of 15–20%. PH is rarely observed without hypoxia during the day. Kessel et al. indicated that the severity of the parameters of the nocturnal event does not affect the presence of PH but showed that a combination of OSA with chronic obstructive pulmonary disease increased the probability of PH. A good method for the treatment of PH in patients with OSA is CPAP, but when it is not enough to correct sleep related hypoxaemia, oxygen supplementary is necessary [25].

Some publications suggest that patients who suffer from obstructive sleep apnea exhibit pulmonary hypertension, which may cause damage, primarily to the right ventricular [26,27,28]. Moreover, patients can present symptoms of right ventricular failure without pulmonary hypertension in some cases. Pulmonary artery pressure may increase during apneas, but the mechanism of disturbances in the right ventricular work is still under discussion. 

Hypoxemia is the most likely cause of the increases in pressure in the pulmonary circulation. It is known that pulmonary vasoconstriction is the consequence of acute hypoxia to maintain an appropriate level of alveolar ventilation. It is a physiological reaction. Intermittent hypoxia is characterized for people with OBS. This state causing pulmonary vasoconstriction may lead to pulmonary hypertension and increases right ventricular afterload.

Patel et al. checked in their research the relationship between diffuse right ventricular fibrosis and heart failure with preserved ejection fraction. The researchers enrolled 14 patients with PH-HFpEF, 13 with pulmonary arterial hypertension, and a control group (eight people). All participants underwent magnetic resonance of the heart to assess myocardial fibrosis. They indicated that the patients with PAH and PH-HFpEF had similar myocardial fibrosis levels, but RV ECV among patients with PH-HFpEF was associated with worse RV function indices-RV free wall strain [29].

Chu et al. assess in their study right ventricular performance and the impact of continuous positive airway pressure therapy in patients with obstructive sleep apnea. Thus, 80 patients with OBS were recruited for the clinical trials. Using 2-dimensional speckle tracking echocardiography and real-time 3-dimensional echocardiography, the researcher assessed the right ventricular function. The obstructive sleep apnea was diagnosed using full night polysomnography. They indicated that patients with diagnosed OBS had significantly decreased global and longitudinal strain, like our study. In their research did not consider the severity of the disease. We also compared the RV free wall (basal, mid, and apical) and the intraventricular septum (basal, mid, and apical) with other polysomnography parameters, and we found a significant positive correlation between OAH and average free wall strain (r = 0.37), snore and mid-free wall strain (r = 0.34), average HR, and basal free wall strain (r = 0.34). Moreover, CSB was positively correlated with basal septal strain and mid septal strain, with r = 0.36 and 0.42, respectively. Chu et al. showed that six-month CPAP therapy caused a significant increase of global and longitudinal strain. Moreover, they observed a decrease of AHI and duration of desaturation <90% [30].

Similar observations were made in research by D’Andrea et al. They indicated that non-invasive ventilation positively affects the function of the right ventricular [31].

Our research showed significant differences between some parameters of right ventricular strain in patients with and without OSA. The strain parameters in patients with OSA were decreased, except for basal free wall strain and apex free wall strain. A similar observation is found in the research Byonauro et al. The study group contained 29 patients with OSA and without heart failure. All participants underwear 3D echocardiography of the right ventricular. The authors showed that patients with OSA like our research had differences in the strain parameters. They did not find differences considering parameters such as TAPSE and the right ventricular fraction between groups with OSA and control [32].

In the next part of the statistical analysis, the correlation between the polysomnographic and echocardiography parameters was checked. The correlation between AHI and the strains was not found in the general study group. AHI was correlated with mild free wall strain only among patients younger than 60 years old. Moreover, the basal septal strain was correlated with AHI only in women.

Altekin et al. contained other conclusions in their publication. The study contained 21 without OSA and 58 individuals with OSA. They used the speckle tracking echocardiography method to check the presence of right ventricular dysfunction before the advent of RV failure and pulmonary hypertension in patients with OSA. They found significant differences between the severity of OSA and the right ventricular strain and systolic strain rate. Moreover, they showed the correlation between strain parameters and the severity of OSA. They indicated like our research that the strain could be a valuable method to detect subclinical right ventricular dysfunction among patients with OSA, even in the absence of pulmonary hypertension [33].

The strength of our study is the use of full night polysomnography in the diagnosis of obstructive sleep apnea. Our research showed a positive correlation between the snore events and mild free wall strain, OAH and average free wall strain, CSB and basal septal strain, average HR, and basal free wall strain. 

There are not many publications containing the relationship between polysomnographic and echocardiographic parameters. Kepez et al. indicated AHI, nocturnal mean SO2, and nocturnal deep desaturation index to be significantly correlated with apical right ventricular peak systolic strain and SR values. Moreover, regression analysis was performed and AHI was an independent predictor of apical right ventricular peak systolic strain. As in our research, there are no correlations between weight, age, and strain parameters [34].

The limitations of our study are the lack of left ventricular strain assessment, lack of information about the coexistence of other respiratory diseases, and no direct assessment of pulmonary artery pressure. 

In the future, similar studies should be considered in subgroups of different sex, age, coexistence of risk factors for cardiovascular diseases, as well as coexistence of other cardiovascular, respiratory, and nervous system diseases.

In summary, among patients with sleep disorders, functional disorders of the right ventricle, assessed using the strain method, are partly observed. Detection of right ventricular dysfunction is critical among patients with OBS in the prevention of heart failure [35].

## Figures and Tables

**Table 1:** Clinical characteristics of the study group.

	X	Me	SD	Min	Max
Age [years]	56.36	59.50	14.77	23.00	81.00
Height [cm]	172.67	173.00	8.96	158.00	188.00
Body mass [kg]	80.27	82.00	19.04	53.00	125.00
Body mass index [kg/m^2^]	26.71	25.75	4.90	19.95	36.13
	n		%		
Age < 60	21		48.8		
Age ≥ 60	22		51.2		
Men	29		67.4		
Women	14		32.5		
Normal body mass	26		60.5		
Overweight/obesity	17		39.5		
Arterial hypertension	26		60.5		
Coronary artery diseases	4		9.3		
Type 2 diabetes	5		11.6		

**Table 2:** Polysomnography parameters in the study group.

	X	Me	SD	Min	Max
AHI [events/hours]	27.09	23.05	19.76	0.20	71.30
ODI [events/hours]	27.44	26.30	19.74	0.20	73.70
Snore [events/hours]	23.43	11.40	24.93	0.00	81.60
OAH [events/hours]	6.84	1.70	10.30	0.00	38.40
CAH [events/hours]	0.80	0.15	1.66	0.00	9.10
CSB [events/hours]	1.60	0.00	4.09	0.00	19.90
Average SpO2 [%]	91.42	92.20	4.75	65.50	96.10
Minimal SpO2 [%]	80.90	82.00	7.45	64.00	94.00
% SpO2 < 90 [%]	14.75	5.30	21.29	0.00	91.00
Average desaturation [%]	6.48	4.50	11.51	2.00	77.00
Average HR [bpm]	61.63	61.85	8.16	45.80	80.00
Minimal HR [bpm]	49.33	50.00	9.31	24.00	69.00
Maximal HR [bpm]	89.85	89.50	15.65	62.00	141.00
Sleep efficiency [%]	78.16	81.25	14.55	25.30	93.60
TST N1 [% sleep time]	8.52	4.50	8.24	0.60	33.90
TST N2 [% sleep time]	54.94	51.60	27.62	24.40	98.00
TST N3 [% sleep time]	21.74	19.35	13.31	1.20	61.50
TST REM [% sleep time]	25.35	21.40	19.28	2.90	98.50
	n		%		
AHI < 5 (without OSA)	10		23.3		
AHI ≥ 5 (OSA)	33		76.7		
AHI: 5–15 (mild OSA)	4		9.3		
AHI: 15–30 (moderate OSA)	15		34.9		
AHI ≥ 30 (severe OSA)	14		32.5		

AHI, apnea–hypopnea index; ODI, oxygen desaturation index; OAH, obstructive apneas events; CAH, central apneas events; CSB, Cheyne-Stokes breathing; SpO2, saturation; HR, heart rate; TST, total sleep time (min); N1, sleep stage 1; N2, sleep stage 2; N3, sleep stage 3; REM, rapid eye movement sleep stage; OSA, obstructive sleep apnea.

**Table 3:** Right ventricular echocardiography parameters in the study group.

	X	Me	SD	Min	Max
RAA [cm^2^]	25.02	24.75	1.13	24.00	26.70
RA major dimension [mm]	49.40	44.00	17.64	24.00	92.00
RA minor dimension [mm]	47.60	43.00	15.93	23.00	84.00
RVOT p [mm]	31.58	32.00	4.33	18.00	40.00
RVOT m [mm]	32.86	33.50	4.45	22.00	41.00
RVOT d [mm]	27.80	27.00	4.03	21.00	37.00
MPA [mm]	26.11	25.00	1.90	24.00	29.00
RVD [mm]	36.95	37.00	4.26	29.00	51.00
s’RV [cm/s]	13.63	13.00	2.46	10.00	20.00
TAPSE [mm]	24.51	24.00	3.60	19.00	34.00
RV E [cm/s]	60.58	60.50	11.67	39.00	92.00
RV A [cm/s]	51.79	47.50	15.74	28.00	109.00
RV E/A	1.00	0.91	0.30	0.71	1.68
RVEDt [ms]	254.42	242.00	64.75	118.00	405.00
RV E’ [cm/s]	10.00	9.00	3.70	4.00	24.00
RV A’ [cm/s]	12.90	13.00	3.45	5.00	21.00
TCO [ms]	393.40	377.00	47.05	319.00	494.00
RVET [ms]	300.93	303.00	27.84	247.00	354.00
RIMP	0.71	0.71	0.15	0.60	0.81
PAV [cm/s]	74.35	73.00	9.88	55.00	104.00
PAAT [ms]	140.93	145.00	26.12	88.00	198.00

RAA, right atrial area; RA, right atrial; RVOT p, proximal right ventricular outflow diameter; RVOT m, mid right ventricular outflow diameter; RVOT d, distal right ventricular outflow diameter; MPA, main pulmonary artery diameter; RVD, basal right ventricle diameter; s’RV, peak systolic velocity of tricuspid annulus by pulsed wave Tissue Doppler Imaging; TAPSE, tricuspid annular plane systolic excursion; RV E, early peak transtricuspid diastolic flow; RV A, late peak transtricuspidal diastolic flow; RV E/A, ratio of early to late peak transtricuspid diastolic flow; RVEDt, deceleration time of early peak transtricuspid diastolic flow; RV E’, early peak diastolic velocity of tricuspid annulus by pulsed wave Tissue Doppler Imaging; RV A’, late peak diastolic velocity of tricuspid annulus by pulsed wave Tissue Doppler Imaging; TCO, tricuspid valve closure opening time; RVET, right ventricular ejection time; RIMP, right ventricular myocardial performance index; PAFV, peak of pulmonary artery flow velocity; PAAT, pulmonary artery acceleration time

**Table 4:** Right ventricular strain parameters in the study group.

	X	Me	SD	Min	Max
Average free wall strain [%]	−27.83	−27.00	5.81	−42.00	−17.50
Basal free wall strain [%]	−27.19	−26.00	6.10	−45.00	−15.00
Mid free wall strain [%]	−28.88	−28.00	5.67	−44.00	−20.00
Apex free wall strain [%]	−23.67	−23.00	6.56	−36.00	−8.00
Average septal strain [%]	−16.58	−17.00	4.41	−25.00	−6.67
Basal septal strain [%]	−16.47	−17.00	4.73	−26.00	−5.00
Mid septal strain [%]	−17.02	−17.00	4.72	−26.00	−4.00
Apex septal strain [%]	−16.26	−15.00	5.60	−29.00	−4.00

**Table 5:** Right ventricular strain parameters in subgroups separated based on OSA (statistically significant differences between the groups were bolded).

	AHI < 5 (without OSA)		AHI ≥ 5 (OSA)		*p* Value
	X	SD	X	SD	
**Average free wall strain** [%]	**−32.64**	**5.40**	**−27.17**	**5.60**	**0.023**
Basal free wall strain [%]	−31.57	7.46	−26.58	5.67	0.052
**Mid free wall strain** [%]	**−33.71**	**3.50**	**−28.30**	**5.65**	**0.020**
Apex free wall strain [%]	−26.71	8.73	−23.03	6.23	0.194
**Average septal strain** [%]	**−20.24**	**3.25**	**−15.93**	**4.21**	**0.015**
**Basal septal strain** [%]	**−20.14**	**3.02**	**−16.06**	**4.55**	**0.030**
**Mid septal strain** [%]	**−20.57**	**2.64**	**−16.48**	**4.74**	**0.034**
**Apex septal strain** [%]	**−20.00**	**5.42**	**−15.24**	**5.26**	**0.037**

**Table 6:** Correlations between polysomnography parameters and right ventricular strain parameters in whole study group (the statistically significant correlation coefficients were bolded).

	Average Free Wall Strain	Basal Free Wall Strain	Mid Free Wall Strain	Apex Free Wall Strain	Average Septal Strain	Basal Septal Strain	Mid Septal Strain	Apex Septal Strain
AHI [events/hours]	0.20	0.08	0.26	0.07	−0.01	0.02	−0.01	−0.04
ODI [events/hours]	0.16	0.04	0.22	0.07	0.05	0.10	0.06	−0.02
Snore [events/hours]	0.19	0.09	**0.34**	0.15	0.03	0.12	0.00	−0.03
OAH [events/hours]	**0.37**	0.21	0.30	0.11	−0.06	−0.17	−0.07	0.05
CAH [events/hours]	0.17	0.18	0.15	−0.01	0.14	0.26	0.24	−0.08
CSB [events/hours]	−0.01	−0.09	0.07	0.11	0.33	**0.36**	**0.42**	0.15
Average SpO2 [%]	−0.28	−0.23	−0.22	−0.14	0.07	0.10	0.11	0.00
Minimal SpO2 [%]	−0.08	−0.01	−0.16	−0.20	−0.14	−0.12	−0.12	−0.12
% SpO2 < 90 [%]	0.17	0.08	0.24	0.19	0.12	0.15	0.12	0.06
Average desaturation [%]	0.11	0.10	0.12	−0.06	−0.07	−0.07	−0.01	−0.11
Average HR [bpm]	0.26	**0.34**	0.20	−0.10	0.17	0.22	0.16	0.09
Minimal HR [bpm]	0.08	0.13	0.02	−0.18	0.02	0.06	0.01	−0.00
Maximal HR [bpm]	0.25	0.24	0.10	0.03	0.19	0.12	0.17	0.20
Sleep efficiency [%]	−0.22	−0.21	−0.25	−0.14	−0.09	−0.07	−0.01	−0.15
TST N1 [% sleep time]	0.20	0.19	0.16	0.07	0.02	−0.07	0.01	0.10
TST N2 [% sleep time]	−0.01	0.02	−0.02	−0.04	0.01	0.05	0.02	−0.03
TST N3 [% sleep time]	0.02	−0.01	0.10	0.11	−0.00	0.05	−0.02	−0.03
TST REM [% sleep time]	0.21	0.07	0.02	−0.07	0.07	0.12	0.12	−0.01

AHI, apnea–hypopnea index; ODI, oxygen desaturation index; OAH, obstructive apneas events; CAH, central apneas events; CSB, Cheyne-Stokes breathing; SpO2, saturation; HR, heart rate; TST, total sleep time (min); N1, sleep stage 1; N2, sleep stage 2; N3, sleep stage 3; REM, rapid eye movement sleep stage; OSA, obstructive sleep apnea.

## Data Availability

The data presented in this study are available upon request from the corresponding author. The data are not publicly available.

## References

[1] Punjabi N.M. (2008). The epidemiology of adult obstructive sleep apnea. Proc. Am. Thorac. Soc..

[2] Khattak H.K., Hayat F., Pamboukian S.V., Hahn H.S., Schwartz B.P., Stein P.K. (2018). Obstructive Sleep Apnea in Heart Failure: Review of Prevalence, Treatment with Continuous Positive Airway Pressure, and Prognosis. Tex. Heart Inst. J..

[3] May A.M., Van Wagoner D.R., Mehra R. (2017). OSA and Cardiac Arrhythmogenesis. Chest.

[4] Pollicina I., Maniaci A., Lechien J.R., Iannella G., Vicini C., Cammaroto G., Cannavicci A., Magliulo G., Pace A., Cocuzza S. (2021). Neurocognitive Performance Improvement after Obstructive Sleep Apnea Treatment: State of the Art. Behav. Sci..

[5] Young T., Finn M.L., Peppard P.E., Szklo-Coxe M., Austin M.D., Nieto F.J., Stubbs B.R., Hla K.M. (2008). Sleep Disordered Breathing and Mortality: Eighteen-Year Follow-up of the Wisconsin Sleep Cohort. Sleep.

[6] Fietze I., Laharnar N., Obst A., Ewert R., Felix S.B., Garcia C., Gläser S., Glos M., Schmidt C.O., Stubbe B. (2019). Prevalence and association analysis of obstructive sleep apnea with gender and age differences—Results of SHIP-Trend. J. Sleep Res..

[7] Pace A., Iannella G., Rossetti V., Visconti I., Gulotta G., Cavaliere C., De Vito A., Maniaci A., Cocuzza S., Magliulo G. (2020). Diagnosis of Obstructive Sleep Apnea in Patients with Allergic and Non-Allergic Rhinitis. Medicina.

[8] Adir Y., Humbert M., Chaouat A. (2021). Sleep-related breathing disorders and pulmonary hypertension. Eur. Respir. J..

[9] Kholdani C., Fares W.H., Mohsenin V. (2015). Pulmonary Hypertension in Obstructive Sleep Apnea: Is it Clinically Significant? A Critical Analysis of the Association and Pathophysiology. Pulm. Circ..

[10] Sajkov D., McEvoy D. (2009). Obstructive Sleep Apnea and Pulmonary Hypertension. Prog. Cardiovasc. Dis..

[11] Sajkov D., Wang T., Saunders N.A., Bune A.J., Neill A.M., Evoy R.D.M.C. (1999). Daytime Pulmonary Hemodynamics in Patients with Obstructive Sleep Apnea without Lung Disease. Am. J. Respir. Crit. Care Med..

[12] De Boer K., Lee J.S. (2015). Under-recognised co-morbidities in idiopathic pulmonary fibrosis: A review. Respirology.

[13] Segers V., Brutsaert D.L., De Keulenaer G. (2012). Pulmonary hypertension and right heart failure in heart failure with preserved left ventricular ejection fraction. Curr. Opin. Cardiol..

[14] Sanner B.M., Doberauer C., Konermann M., Sturm A., Zidek W. (1997). Pulmonary Hypertension in Patients with Obstructive Sleep Apnea Syndrome. Arch. Intern. Med..

[15] Li J., Lin X., Li H., Lu C., Li R., Liu W., Wang Z. (2020). Right ventricular diastolic dysfunction in patients with obstructive sleep apnea syndrome. Echocardiography.

[16] Uematsu M. (2010). Is Right Ventricular Dysfunction in Obstructive Sleep Apnea a Potential Screening Tool?. Circ. J..

[17] Tugcu A., Yildirimtürk O., Tayyareci Y., Demiroglu C., Aytekin S. (2010). Evaluation of Subclinical Right Ventricular Dysfunction in Obstructive Sleep Apnea Patients Using Velocity Vector Imaging. Circ. J..

[18] Zakhama L., Herbegue B., Abouda M., Antit S., Slama I., Boussabah E., Thameur M., Masmoudi M., Abdelaali N., Charfi M.R. (2016). Impact of obstructive sleep apnea on the right ventricle. Tunis Med..

[19] Collier P., Phelan D., Klein A. (2017). A Test in Context: Myocardial Strain Measured by Speckle-Tracking Echocardiography. J. Am. Coll. Cardiol..

[20] Berry R.B., Budhiraja R., Gottlieb D.J., Gozal D., Iber C., Kapur V.K., Marcus C.L., Mehra R., Parthasarathy S., Quan S.F. (2012). Rules for Scoring Respiratory Events in Sleep: Update of the 2007 AASM Manual for the Scoring of Sleep and Associated Events. Deliberations of the Sleep Apnea Definitions Task Force of the American Academy of Sleep Medicine. J. Clin. Sleep Med..

[21] Loredo J.S., Ziegler M.G., Ancoli-Israel S., Clausen J.L., Dimsdale J.E. (1999). Relationship of Arousals From Sleep to Sympathetic Nervous System Activity and BP in Obstructive Sleep Apnea. Chest.

[22] Vasheghani-Farahani A., Kazemnejad F., Sadeghniiat-Haghighi K., Saadat S., Poor P.T., Yazdani T., Alidoosti M., Amooeian V.G., Ashraf H. (2018). Obstructive sleep apnea and the severity of coronary artery disease. Casp. J. Intern. Med..

[23] Patel N., Donahue C., Shenoy A., Patel A., El-Sherif N. (2017). Obstructive sleep apnea and arrhythmia: A systemic review. Int. J. Cardiol..

[24] Coniglio A.C., Mentz R.J. (2020). Sleep Breathing Disorders in Heart Failure. Heart Fail. Clin..

[25] Kessler R., Chaouat A., Weitzenblum E., Oswald M., Ehrhart M., Apprill M., Krieger J. (1996). Pulmonary hypertension in the obstructive sleep apnoea syndrome: Prevalence, causes and therapeutic consequences. Eur. Respir. J..

[26] Nokes B., Raza H., Malhotra A. (2020). Pulmonary hypertension and obstructive sleep apnea. J. Clin. Sleep Med..

[27] Mesarwi O., Malhotra A. (2020). Obstructive sleep apnea and pulmonary hypertension: A bidirectional relationship. J. Clin. Sleep Med..

[28] Wang S., Cui H., Ji K., Ren C., Guo H., Zhu C., Lai Y., Wang S. (2020). Effect of obstructive sleep apnea on right ventricular ejection fraction in patients with hypertrophic obstructive cardiomyopathy. Clin. Cardiol..

[29] Patel R., Li E., Benefield B.C., Swat S.A., Polsinelli V.B., Carr J.C., Shah S., Markl M., Collins J.D., Freed B.H. (2020). Diffuse right ventricular fibrosis in heart failure with preserved ejection fraction and pulmonary hypertension. ESC Heart Fail..

[30] Chu A.-A., Yu H.-M., Yang H., Tian L.-M., Hu Z.-Y., Jiang N., Xie W.-X., Huang Y. (2020). Evaluation of right ventricular performance and impact of continuous positive airway pressure therapy in patients with obstructive sleep apnea living at high altitude. Sci. Rep..

[31] D’Andrea A., Martone F., Liccardo B., Mazza M., Annunziata A., Di Palma E., Conte M., Sirignano C., D’Alto M., Esposito N. (2016). Acute and Chronic Effects of Noninvasive Ventilation on Left and Right Myocardial Function in Patients with Obstructive Sleep Apnea Syndrome: A Speckle Tracking Echocardiographic Study. Echocardiography.

[32] Buonauro A., Galderisi M., Santoro C., Canora A., Bocchino M.L., Lo Iudice F., Lembo M., Esposito R., Castaldo S., Trimarco B. (2017). Obstructive sleep apnoea and right ventricular function: A combined assessment by speckle tracking and three-dimensional echocardiography. Int. J. Cardiol..

[33] Altekin R.E., Karakas M.S., Yanikoglu A., Ozel D., Ozbudak O., Demir I., Deger N. (2012). Determination of right ventricular dysfunction using the speckle tracking echocardiography method in patients with obstructive sleep apnea. Cardiol. J..

[34] Kepez A., Niksarlioglu E.Y.O., Hazirolan T., Ranci O., Kabul H.K., Demir A.U., Kaya E.B., Kocabas U., Aytemir K., Sahin A. (2009). Early Myocardial Functional Alterations in Patients with Obstructive Sleep Apnea Syndrome. Echocardiography.

[35] Tadic M., Cuspidi C., Grassi G., Mancia G. (2021). Obstructive sleep apnea and cardiac mechanics: How strain could help us?. Heart Fail. Rev..

